# Supplementation of hyaluronic acid injections with vitamin D improve knee function by attenuating synovial fluid oxidative stress in osteoarthritis patients with vitamin D insufficiency

**DOI:** 10.3389/fnut.2023.1026722

**Published:** 2023-04-04

**Authors:** Jianlin Shen, Xiaoning Lin, Yujin Lin, Jiecheng Xiao, Changfu Wu, Feng Zheng, Xianwei Wu, Haibin Lin, Guoli Chen, Huan Liu

**Affiliations:** Department of Orthopedics, Affiliated Hospital of Putian University, Putian, Fujian, China

**Keywords:** vitamin D, hyaluronic acid, synovial fluid, oxidative stress, osteoarthritis

## Abstract

**Objectives:**

There is still controversy about the effect of vitamin D supplementation on osteoarthritis (OA). The purpose of this study was to investigate the effects of vitamin D supplementation with Hyaluronic acid (HA) injection on OA.

**Methods:**

We investigated serum vitamin D levels and oxidative stress (OS) in synovial fluid from patients with OA who underwent total knee arthroplasty (grade IV, *n* = 24) and HA injection (grade II and III, *n* = 40). The effects of HA injection with or without oral vitamin D supplementation on synovial fluid OS and knee pain and function were then further investigated. Finally, patients underwent HA injection were divided into two groups according to vitamin D levels (vitamin D < or > 30 ng/ml), and the efficacy of the two groups were compared.

**Results:**

The results showed that the levels of glutathione peroxidase (GSH-PX) (*P* < 0.05) in the synovial fluid were lower in patients with stage IV OA than that in patients with stage II-III OA, while the levels of malondialdehyde (MDA) (*P* < 0.05) and lactate dehydrogenase (LDH) (*P* < 0.01) were significantly higher. Moreover, we found that age, BMI and vitamin D levels were significantly associated with the levels of oxidants and/or antioxidants in synovial fluid, and that vitamin D was significantly negatively correlated with BMI (*R* = −0.3527, *p* = 0.0043). Supplementation of HA injections with vitamin D significantly reduced the OS status in synovial fluid, attenuated knee pain and improved knee function in OA patients with vitamin D insufficiency.

**Conclusion:**

We conclude that maintenance of vitamin D sufficiency may be beneficial for the treatment of OA by improving OS in synovial fluid.

## 1. Introduction

Osteoarthritis (OA), one of the most common chronic diseases in elderly individuals, primarily affects weight bearing synovial joints (1). Historically, only degeneration and destruction of articular cartilage was assumed contribute to the progression of OA, but it is now known that all tissues within the joint structure are involved, including alterations in synovial membrane, meniscus, ligaments, subchondral bone and infrapatellar fat pad (2, 3). Moreover, OA is a multifactorial disease with systemic and local factors being involved such as age, sex, obesity, physical activity, previous joint trauma, occupation, etc. (4). Oxidative stress (OS), a series of adaptive responses caused by the imbalance between reactive oxygen species (ROS) production and antioxidant systems, is also considered as potential OA disease culprits (5, 6). Excessive ROS production can lead to mitochondrial dysfunction, interfere with cartilage homeostasis and promote catabolism (7) and subsequent destruction of many components of the joint, including collagen, proteoglycans, and hyaluronic acid (8).

Lactate dehydrogenase (LDH) is an important enzyme in anaerobic glycolysis and has a pro-ROS forming function in chondrocytes (9). Malondialdehyde (MDA), the end byproduct of lipid peroxidation during OS, has been widely used as an oxidative biomarker in previous OA studies (8, 10). Fortunately, there exist several antioxidant enzymes that protect joint tissues against damage from ROS, such as superoxide dismutase (SOD) and glutathione peroxidase (GSH-Px) (8, 11).

Vitamin D is a steroid hormone precursor that is important for many joint structures, such as cartilage, subchondral bone and muscle tissue, all of which contribute to the progression of knee OA (12). More than 50% of patients with primary knee OA are vitamin D deficient (13) and lower levels of 25-hydroxyvitamin D [25(OH)D] were shown to be associated with poor quadriceps function, increased knee pain and OA progression (14, 15). However, several randomized controlled trials of vitamin D supplementation in OA have yielded conflicting results (13, 16, 17). A study by Manoy et al. (18) highlighted the role of vitamin D as an antioxidant in dietary supplementation for OA. Vitamin D is one of the key controllers of oxidative stress, mitochondrial respiratory function and systemic inflammation, and thus, the aging process in humans (19). Hypovitaminosis D impairs mitochondrial functions and enhances OS. Conversely, maintaining adequate vitamin D reduces OS and improves mitochondria functions (19). Therefore, it is not surprising that vitamin D deficiency may increase the incidence and severity of several common age-related diseases, such as metabolic disorders related to oxidative stress (19).

Hyaluronic acid (HA) is an important part of synovial fluid and the cartilage matrix, being responsible for lubricating joints, resisting infection, and participating in cartilage repair (20). HA intracavitary injection is currently widely used as a treatment relieving joint pain in OA (21). Several studies support the fact that HA can reduce ROS levels and protect chondrocytes from OS damage (22–24). In addition, vitamin D has been proposed to have antioxidant properties and vitamin D supplementation can mitigate ROS production, augment antioxidant capacity, and prevent OS (18, 25, 26). Synovial fluid is an accessible fluid that represents biological processes within the joint and more accurately reveals changes in joint tissue (27). However, few studies have investigated the effect of vitamin D supplementation on OS status in the synovial fluid of individuals with OA. Therefore, this study was performed to evaluate the OS status in the synovial fluid of OA patients with different grades of joint degeneration. Furthermore, the effects of HA injection with or without oral vitamin D supplementation on synovial fluid OS and knee pain and function were also investigated.

## 2. Materials and methods

### 2.1. Study design

The study consisted of two parts. First, the OS levels in synovial fluid from patients with different degrees of joint degeneration were compared, and the relationship between serum vitamin D levels and OS in synovial fluid was observed. Second, a randomized controlled trial was performed to compare the effects of HA injection alone or in combination with vitamin D supplementation on knee function and OS levels in synovial fluid.

### 2.2. Participants

From June 2021 to January 2022, a total of 24 patients with stage IV OA undergoing total knee arthroplasty and 40 patients with stage II-III OA undergoing HA injection were investigated. Patients were eligible if they (1) aged > 40 years, (2) primary OA, (3) had radiological evidence of knee OA (4) knee pain for most days of the previous month. The exclusion criteria were as follows: (1) secondary OA, (2) inflammatory arthritis, such as rheumatoid arthritis, ankylosing spondylitis, (3) use of cod liver oil or vitamin supplementation containing vitamin D, glucosamine or chondroitin within 3 months, (4) diseases affecting bone metabolism, such as hyperparathyroidism, chronic liver and kidney insufficiency, and chronic intestinal diseases, such as chronic diarrhea, (5) other diseases of the joints, such as tuberculosis, tumors; and (6) Kellgren-Lawrence grading of OA (28) was 0 or I. Written informed consent was obtained from each subject and ethical approval was given by ethics committee of Affiliated Hospital of Putian University (202009).

### 2.3. Randomization and blinding

Patients receiving HA injection were randomly divided into two groups of 20 patients each. Group 1 was HA injection alone, while group 2 was HA injection combined with oral vitamin D. Randomization and drug assignment in this study were performed by a non-study team (professional nurses). According to the dosage in the instructions, ARTZ Dispo (Sodium Hyaluronate Injection; Seikagaku Corporation, Tokyo, Japan) was used for intracavitary injection once a week for 5 consecutive weeks, and Calcitriol (Roche Corporation, Shanghai, China) was used for vitamin D supplementation at a dose of 0.25 μg twice a day for 3 months (29). To maximize the effect of vitamin D on bone and calcium metabolism, serum 25-hydroxyvitamin D levels should be more than 30 ng/ml. Therefore, we performed a further comparative analysis of two groups based on vitamin D levels, where vitamin D < 30 ng/ml was defined as insufficient (30).

### 2.4. Outcome measures

Knee radiographs and blood samples from OA patients were taken before the total knee arthroplasty (TKA) and HA injection, while synovial fluid was collected during TKA and the first and fifth HA injection, respectively. Levels of vitamin D (25-OH-D3) were directly quantified on an instrument by electrochemiluminescence (Cobase 601, ROCH, Basel, Switzerland). In synovial fluid, the detection kits were used to determinate the levels of total SOD (A001), MDA (A003) and GSH-Px (A005) according to the manufacturer’s instructions (Nanjing Jiancheng Bioengineering Institute, Nanjing, China). The levels of LDH in synovial fluid were assessed by oxidase method (BIOBASE, Shandong, China).

A visual analogue scale (VAS) (31) was used to assess pain intensity. The scale was a 10-cm line with two endpoints representing the extreme states “no pain” (0) and “the maximal pain imaginable” (10). The standard of pain degree as follows: mild pain, 0–3 points; moderate pain, 4–6 points; severe pain, 7–10 points. The patients marked the horizontal line according to their self-perception to indicate the degree of pain. The Western Ontario and McMaster Universities Osteoarthritis Index (WOMAC) (32) questionnaire was used to assess OA disability. The WOMAC is composed of 24 items divided into 3 aspects: functional pain (5 items), stiffness (2 items), and difficulty in activities of daily living (17 items). Higher values indicate poorer WOMAC subscale scores for pain and physical function. The questionnaires (VAS and WOMAC) were administered at baseline, 5 weeks and 3 months.

### 2.5. Statistical analysis

Data were given as mean ± SD for normally distributed variables. Ratio was used to describe categorical variables. The normality of the data distribution was tested by the one-sample Kolmogorov–Smirnov test. The Student’s *t*-test was applied in normally distributed variables, while the Mann–Whitney U test was used for non-normally distributed variables. Categorical variables were analyzed using the chi-square test. The Pearson’s correlation coefficient test was used for the correlation of variables. The *P*-value reported was two-sided and a value of *P* < 0.05 was considered statistically significant. All data analysis were performed using the GraphPad Prism software (version 9.0, GraphPad Software, San Diego, CA, USA).

## 3. Results

### 3.1. The demographic data of patients

The comparative demographic data between the patients with stage IV OA undergoing total knee arthroplasty and patients with stage II-III OA undergoing HA injection are shown in Table 1. There was a significant difference in age (*P* < 0.0001), and no significant differences were found in sex, weight, height, BMI and serum vitamin D levels. Patients receiving HA injection were randomly divided into two groups of 20 patients each. Group 1 was HA injection alone, while group 2 was HA injection combined with oral vitamin D. The comparative demographic data between the two groups are shown in Table 2, and no significant differences were found in any indicators.

**TABLE 1:** Demographic data of all patients.

	OA (II-III)	OA (IV)	*t* value	*p*-value
Number of patients	40	24		
Age (years)	56.38 ± 6.335	68.88 ± 6.674	7.490	<0.0001
Gender (male/female)	10/30	7/17		0.7744
Weight (kg)	61.18 ± 6.567	61.46 ± 5.421	0.1779	0.8593
Height (cm)	160.9 ± 6.514	161.1 ± 6.406	0.09472	0.9248
BMI (kg/m^2^)	23.58 ± 1.528	23.69 ± 1.777	0.2705	0.7877
Vitamin D (ng/mL)	34.97 ± 7.841	31.87 ± 9.109	1.439	0.1551

**TABLE 2:** Demographic data of HA-injected patients with or without vitamin D supplementation.

	Group 1	Group 2		
	**HA**	**HA + vitamin D**	***t* value**	***p*-value**
Number of patients	20	20		
Age (years)	58.10 ± 7.115	54.65 ± 5.050	1.768	0.085
Gender (male/female)	4/16	6/14		0.4652
Weight (kg)	60.40 ± 5.942	61.95 ± 7.207	0.7421	0.4626
Height (cm)	160.2 ± 6.343	161.7 ± 6.752	0.7482	0.4589
BMI (kg/m^2^)	23.52 ± 1.308	23.65 ± 1.758	0.2671	0.7909
Vitamin D (ng/mL)	34.10 ± 6.974	35.34 ± 8.744	0.4968	0.6222

Data are expressed as mean ± standard deviation or *n*/*n*. OA, osteoarthritis; BMI, body mass index; HA, hyaluronic acid. *t*-value represents the ratio of the difference between two groups to the difference within the groups.

### 3.2. Oxidative stress levels in synovial fluid between OA patients with different grades of joint degeneration

As shown in Table 3, the level of SOD in synovial fluid was lower in patients with stage IV OA than in patients with stage II-III OA, but the difference was not significant (*P* = 0.0505). However, the GSH-Px level was significantly lower (*P* < 0.05), and levels of MDA (*P* < 0.05) and LDH (*P* < 0.01) were significantly higher in patients with stage IV OA.

**TABLE 3:** OS levels in synovial fluid between patients with stage II-III OA and stage IV OA.

	OA (II-III)	OA (IV)	*t*-value	*p*-value
SOD (U/mL)	8.669 ± 1.169	8.022 ± 1.392	1.994	0.505
GSH-Px (nmol/min/mL)	111.8 ± 18.72	100.8 ± 20.62	2.195	<0.05
MDA (nmol/mL)	5.663 ± 1.304	6.366 ± 1.215	2.140	<0.05
LDH (U/L)	118.4 ± 49.99	164.3 ± 71.37	3.019	<0.01

Data are expressed as mean ± standard deviation. OA, osteoarthritis; SOD, superoxide dismutase; GSH-Px, glutathione peroxidase; MDA, malondialdehyde; LDH, lactate dehydrogenase. *t*-value represents the ratio of the difference between two groups to the difference within the groups.

### 3.3. Correlation analysis of the differences between demographic data, vitamin D levels, and OS

Correlation analysis of the differences between age, sex, BMI, serum vitamin D levels and OS in synovial fluid was performed in all patients. The results are shown in Table 4. Age was significantly correlated with OS in synovial fluid, which was negatively correlated with SOD (*p* = 0.05) and GSH-Px (*p* = 0.0119) levels and positively correlated with MDA (*p* = 0.0077) and LDH (*p* = 0.001) levels. BMI was found to be significantly negatively correlated with vitamin D (*p* = 0.0043) and SOD (*p* = 0.0237) levels, and positively correlated with MDA levels (*p* = 0.0391). In addition, vitamin D levels were also shown to be significantly negatively correlated with MDA (*p* = 0.037) and LDH (*p* = 0.0436) levels.

**TABLE 4:** Correlation analysis of the differences between demographic data, vitamin D levels, and OS.

		Vitamin D	SOD	GSH-Px	MDA	LDH
Age	R	−0.1351	−0.2461	−0.3126	0.3301	0.4015
	P	0.2872	0.05*	0.0119*	0.0077**	0.001**
Gender	R	−0.1612	0.05934	0.06583	−0.1277	−0.1360
	P	0.2032	0.6414	0.6053	0.3145	0.2840
BMI	R	−0.3527	−0.2825	−0.1771	0.2585	0.2125
	P	0.0043**	0.0237*	0.1615	0.0391*	0.0918
Vitamin D	R	1	0.2415	0.03473	−0.2613	−0.2531
	P	–	0.0545	0.7853	0.037*	0.0436*

*R* = Pearson correlation, *P* = *p*-value. *p < 0.05, **p < 0.01. BMI, body mass index; SOD, superoxide dismutase; GSH-Px, glutathione peroxidase; MDA, malondialdehyde; LDH, lactate dehydrogenase.

### 3.4. Effects of vitamin D supplementation combined with HA injection on OS in synovial fluid

**FIGURE 1:**
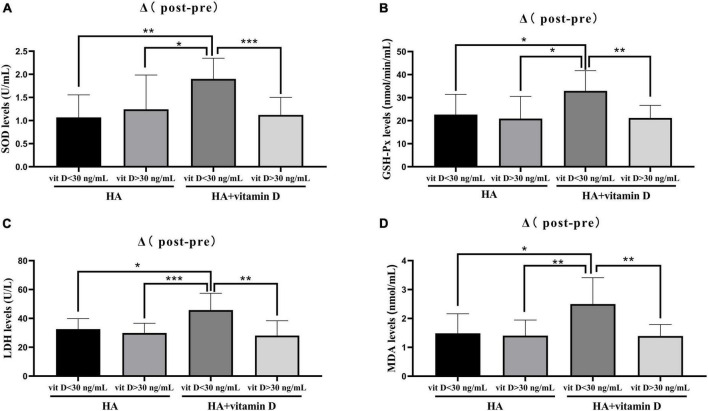
Changes of OS levels in groups with different vitamin D levels. **(A)** Change in SOD level after 5 weeks of treatment; **(B)** Change in GSH-Px level after 5 weeks of treatment; **(C)** Change in LDH level after 3 months of treatment; **(D)** Change in MDA level after 3 months of treatment. *p < 0.05, **p < 0.01, ***p < 0.001.

Patients undergoing HA injection were randomly divided into two groups of 20 patients each. Group 1 was HA injection alone, while group 2 was HA injection combined with oral vitamin D. After 5 weeks of treatment, the levels of OS in both groups were significantly improved, among which levels of the antioxidative agents SOD (group 1: *p* = 0.0063, group 2: *P* = 0.0002) and GSH-Px (group 1: *p* = 0052, group 2: *P* < 0.0001) significantly increased, while those of the oxidative agents MDA (group 1: *p* = 0021, group 2: *P* < 0.001) and LDH (group 1: *p* = 0293, group 2: *P* = 0.0253) were significantly decreased (Table 5). However, there were no statistically significant differences in synovial fluid SOD, GSH-Px, MDA and LDH levels between the two groups in terms of pre-treatment, post-treatment and Δ (post-pre) (Table 5). Moreover, we performed a further comparative analysis of two groups according to the vitamin D level (30 ng/ml), in which patients with vitamin D insufficiency accounted for 35% (7/20) in group 1 and 40% (8/20) in group 2. Interestingly, we found that patients with vitamin D insufficiency in group 2 shown the most significant changes in OS between pre- and post-treatment (5 weeks) (Figure 1).

**TABLE 5:** Effect of HA injection with or without vitamin D supplementation on synovial fluid OS.

	Group 1	Group 2		
	**HA**	**HA + vitamin D**	***t* value**	***p*-value**
**SOD (U/mL)**				
Pre-treatment	8.660 ± 1.269	8.677 ± 1.092	0.0448	0.9645
Post-treatment (5 weeks)	9.841 ± 1.315**	10.11 ± 1.067***	0.7105	0.4818
Δ (post-pre) (5 weeks)	1.181 ± 0.6565	1.433 ± 0.5571	1.310	0.1981
**GSH-Px (nmol/min/mL)**				
Pre-treatment	114.1 ± 20.58	109.6 ± 16.89	0.7590	0.4526
Post-treatment (5 weeks)	135.6 ± 25.12**	135.4 ± 16.4***	0.0218	0.9827
Δ (post-pre) (5 weeks)	21.51 ± 9.174	25.88 ± 8.977	1.523	0.1359
**MDA (nmol/mL)**				
Pre-treatment	5.587 ± 1.42	5.739 ± 1.21	0.3648	0.7173
Post-treatment (5 weeks)	4.154 ± 1.324**	3.904 ± 1.212***	0.6229	0.5371
Δ (post-pre) (5 weeks)	1.433 ± 0.5736	1.835 ± 0.8412	1.767	0.0853
**LDH (U/L)**				
Pre-treatment	120.3 ± 52.85	116.6 ± 48.25	0.2343	0.8160
Post-treatment (5 weeks)	88.25 ± 34.82*	83.65 ± 40.76*	0.3837	0.7033
Δ (post-pre) (5 weeks)	32.05 ± 20.37	32.90 ± 13.76	0.1546	0.8779

*Represents the comparison between pre- and post-treatment (5 weeks). *p < 0.05, **p < 0.01, ***p < 0.001. HA, hyaluronic acid; SOD, superoxide dismutase; GSH-Px, glutathione peroxidase; MDA, malondialdehyde; LDH, lactate dehydrogenase. *t*-value represents the ratio of the difference between two groups to the difference within the groups.

### 3.5. Effects of vitamin D supplementation combined with HA injection on knee pain and function

**FIGURE 2:**
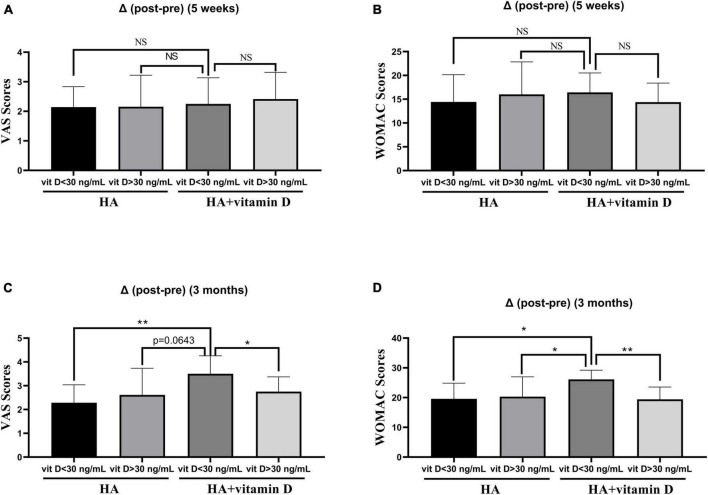
Changes of the VAS and WOMAC scores in groups with different vitamin D levels. **(A)** Change in the VAS score after 5 weeks of treatment; **(B)** Change in the WOMAC score after 5 weeks of treatment; **(C)** Change in the VAS score after 3 months of treatment; **(D)** Change in the WOMAC score after 3 months of treatment. *p < 0.05, **p < 0.01, NS represents no significant.

As shown in Table 6, the VAS and WOMAC scores changed significantly at 5 weeks and 3 months of treatment in both groups (*P* < 0.05). However, no significant differences were found in the VAS or WOMAC scores between the two groups in terms of pre-treatment, post-treatment (5 weeks) and post-treatment (3 months). Similar to the abovementioned changes in OS, there were no differences in the VAS and WOMAC scores at 5 weeks after treatment, while at 3 months after treatment, the changes in the VAS and WOMAC scores of patients with vitamin D insufficiency (<30 ng/ml) in group 2 were significantly higher than those of patients in the other groups (Figure 2).

**TABLE 6:** Effect of HA injection with or without vitamin D supplementation on VAS and WOMAC Scores.

	HA	HA + vitamin D	*t* value	*p*-value
**VAS Pain (0–10 points)**				
Pre-treatment	5.75 ± 1.118	5.85 ± 1.040	0.2929	0.7712
Post-treatment (5 weeks)	3.65 ± 0.8127*	3.55 ± 0.7592*	0.4021	0.6898
Post-treatment (3 months)	3.25 ± 0.7164*	2.80 ± 0.8944*^#^	1.756	0.0871
*F* value	44.64	61.66		
*p*-value	<0.0001	<0.0001		
**WOMAC (0–100 points)**				
Pre-treatment	47.65 ± 8.362	48.30 ± 5.516	0.2902	0.7733
Post-treatment (5 weeks)	33.20 ± 5.493*	33.20 ± 4.479*	0.631	0.5318
Post-treatment (3 months)	27.60 ± 5.394*^#^	26.20 ± 4.137*^#^	0.921	0.3628
*F* value	51.23	113.2		
*p*-value	<0.0001	<0.0001		

*Represents the comparison between pre- and post-treatment; ^#^represents the comparison between post-treatment (5 weeks) and post-treatment (3 months). HA, hyaluronic acid; WOMAC, Western Ontario and McMaster Osteoarthritis Index. Scored 0–100, 100 worst; VAS, visual analog scale. Scored 0–100, 10 worst.

## 4. Discussion

Increasing evidence has suggested that OS plays an important role in the pathogenesis of OA (5, 33–36). However, inconsistent results have been obtained as to whether there is an association between the grade of OA and the level of OS in synovial fluid (8, 11, 36, 37). The lack of consistency in results from different studies may be explained by a bias in OA sample selection and the presence of other factors that affect OS, such as inflammation (33, 38). In this study, we found that there were higher levels of oxidative agents (LDH and MDA) and lower levels of antioxidative agents (GSH-Px) in synovial fluid from OA patients with stage IV than in that from patients with stage II-III. The results demonstrated that the level of OS in synovial fluid may increase with the development of OA.

Many risk factors are known to be closely related to the development of OA, such as age, sex, obesity (4), and vitamin D deficiency (12). Therefore, we also assessed the correlation of these factors with OS in synovial fluid. The results showed that age, obesity, and vitamin D levels are associated with the levels of oxidants and/or antioxidants in synovial fluid, suggesting that these risk factors may contribute to the development of OA, at least in part, by regulating OS in synovial fluid. Among them, the most prominent risk factor is an increasing age. Our own results in the current study showed that patients with grade IV OA were older and had higher OS levels in synovial fluid than those with grade II-III OA, and that OS levels in synovial fluid also increased with increasing age, suggesting a certain link among age, OS in synovial fluid and the development of OA. As a degenerative factor, age-related mitochondrial dysfunction leads to an imbalance between ROS production and cellular antioxidant capacity, eventually leading to oxidative damage, which is a hallmark of OA (39–42). The effects of insufficient oxidant scavenging in joints may lead to metalloproteinase activation and direct oxidative damage to structural extracellular matrix proteins in cartilage, and depolymerization of hyaluronate in synovial fluid leading to changes in synovial fluid viscosity, ultimately leading to OA-related cartilage matrix dysregulation and tissue loss (36). In addition, we found a significant negative correlation between vitamin D levels and BMI, which was consistent with previous studies (43–45). It is hypothesized that obesity leads to reduced vitamin D bioavailability due to higher sequestration and lower mobilization of vitamin D by excess adipose tissue (46, 47). Volumetric dilution of vitamin D in larger adiposity mass has also been determined (48). Alternatively, obesity may reduce bioavailability of vitamin D by inhibiting hepatic enzyme 25-hydroxylation of vitamin D to 25-OHD (49). In addition, the following reasons may also explain the low vitamin D status in obesity: (1) lower sun exposure; (2) reduced hepatic synthesis of 25(OH)D due to obesity-associated secondary hyperparathyroidism; (3) negative feedback from an increased 1,25(OH)D concentration; (4) VDR polymorphisms; (5) lower prevalence of vitamin D supplement use (45, 50, 51).

HA intracavitary injection is still one of the most common treatment methods for early- and mid-stage OA (52). HA can attenuate inflammatory events by stimulating the production and accumulation of proteoglycans (53). Previous reports revealed that HA injection reduced markers of inflammation and apoptosis by inhibiting the OS of neutrophils in the synovial fluid of OA patients (24). In the present study, we found that HA injection reduced oxidants levels and increased antioxidant levels in synovial fluid from OA subjects while attenuating knee pain and improving knee function. Therefore, in addition to its lubricating effect, HA can also reduce OS in synovial fluid, thereby achieving the therapeutic effect of OA.

There is still controversy about the effect of vitamin D supplementation on OA (54). Whether vitamin D acts locally on OA articular cartilage or through the body’s endocrine system remains unclear. Although vitamin D supplementation may have a preventive effect on joint pain, there is little evidence from clinical observations that vitamin D has a protective effect on cartilage volume reduction or initiation of OA radiation (15). In this study, we found that supplementation of HA injections with vitamin D did not have a significantly effect on OS levels in synovial fluid or joint pain and function. However, in OA patients with insufficient vitamin D levels (<30 ng/ml), supplementation HA injections with vitamin D (5 weeks) could significantly reduce the OS levels in synovial fluid, which in turn attenuates knee pain and improves knee function after 3 months of HA injection. This is consistent with previous studies showing that patients with insufficient vitamin D levels may benefit from vitamin D supplementation for joint pain relief (13, 15, 55–58). Vitamin D can be regarded as an antioxidant, and vitamin D deficiency can lead to mitochondrial dysfunction, increased ROS production, and oxidative damage (18). A recent meta-analysis of clinical trials showed that vitamin D supplementation improved the serum levels of nitric oxide, SOD, and total antioxidant capacity, while reducing serum level of 8-hydroxydeoxyguanosine, MDA, and myeloperoxidase enzyme (59).

There are several limitations in our study. Due to the invasive procedure, we did not perform detection of OS levels in synovial fluid for patients treated for 3 months.

## 5. Conclusion

Taken together, our results showed that OS levels in synovial fluid increased with the development of OA. Moreover, we found that age, obesity and vitamin D levels may contribute to the development of OA, at least in part, by impacting OS levels in synovial fluid. Last and foremost, supplementation HA injections with vitamin D significantly reduced OS levels in synovial fluid, attenuated knee pain and improved knee function in OA patients with vitamin D insufficiency. Based on previous studies and our results, we conclude that for OA patients with vitamin D deficiency, vitamin D supplementation should be beneficial in addition to primary therapy, possibly through improved OS in synovial fluid.

## Data availability statement

The original contributions presented in this study are included in the article/supplementary material, further inquiries can be directed to the corresponding authors.

## Ethics statement

The studies involving human participants were reviewed and approved by the Ethics Committee of Affiliated Hospital of Putian University. The patients/participants provided their written informed consent to participate in this study.

## Author contributions

JS, GC, HL, and XL conceptualized the idea, performed part of the experiments, analyzed and interpreted all data, and drafted the manuscript. YL, JX, CW, and FZ contributed to sample collection and patient recruitment. XW and HBL surgeon for THA surgery. All authors gave the final approval of the manuscript.
